# Brucellosis presenting as pyrexia of unknown origin in an international traveller: a case report

**DOI:** 10.4076/1757-1626-2-7969

**Published:** 2009-09-01

**Authors:** Vijay Hadda, GC Khilnani, Saurabh Kedia

**Affiliations:** Department of Medicine, All India Institute of Medical SciencesNew Delhi, 110029India

## Abstract

**Introduction:**

In this era of globalization frequent traveling across the world is common. It has resulted in exchange of knowledge and expertise among medical professionals around the world which has a visible positive impact. However, this predisposes the travelers to the risk of acquiring an ‘alien’ disease endemic to a particular country. This may be a great challenge for the treating physicians to manage such patients due to lack of facility for diagnosis and experience in handling such disease. We present a similar case scenario and problems we faced in managing that patient.

**Case presentation:**

A 40-year-old man visited to Africa, developed a skin rash over ankle after an insect bite. This was followed by high grade fever. He was investigated in Kenya, however, returned to India pending results. Later he developed sleepiness and coarse tremors. Work up for the cause of fever was inconclusive. He was diagnosed with trypanosomiasis based on reports from Kenya. In absence of alternate diagnosis and clinical setting, we treated him for trypanosomiasis. This therapy resulted no improvement in patient’s condition. Finally, at request of the patient’s attendants he was referred to Belgium where he was diagnosed as brucellosis and treated successfully.

**Conclusion:**

Our patient was indeed suffering from neurobrucellosis. Brucellosis is a frequently missed cause of pyrexia. Our case highlights that in this era, taking help from our professional colleagues over the globe is easy which can improve patient care greatly.

## Introduction

Globalization has affected all professionals around the world including medical professionals significantly. It has a great positive impact on medical professionals around the world. It has resulted in exchange of knowledge and expertise among medical professionals around the world which has visible positive impact. However, this predisposes the travellers to risk of acquiring an ‘alien’ disease endemic in that country. Lack of facility for diagnosis and experience in handling that particular disease may be a great challenge. We present a similar case scenario where a patient was admitted in India with probable diagnosis of human African trypanosomiasis (HAT). We discuss the case and problems faced by us managing this patient.

## Case presentation

A forty-year Indian, working in pharmaceutical company, had gone on a business trip to Africa traveling through Uganda, Tanzania and Kenya. While he was in Africa, he developed a swelling over the left ankle joint after an insect bite. This swelling was painful and followed by high grade fever with chills and rigors after 3-4 days. There was no history of any joint pain. There was no other clue on history to localize the cause of fever. There was no history of high risk behavior. Patient had past history of essential tremors for last 6 years, without any functional disability. There was no history of hypertension, diabetes, stroke, or tuberculosis in the past. He took empirical antibiotics in Africa which resulted minor relief in his symptoms. He consulted a physician at Kenya, who suggested blood investigations. However, patient had to return to India because of personal reasons, pending his investigations result. On examination, he was febrile with oral temperature of 102 F. He had tachycardia with pulse rate of 116/min, blood pressure was 124/80 mm of Hg. There was an erythematous swelling over the left ankle, with peri-lesional erythematous rashes. There was no other skin rash elsewhere. There was no pallor, icterus, or peripheral lymphnode enlargement. Examination of respiratory and cardiovascular system was normal. Abdomen examination revealed hepatomegaly of 3 cm and splenomegaly of 3 cm below costal margin. Central nervous system examination revealed coarse tremors in bilateral hands. Other motor, sensory and cranial nerves examination was normal. There were no signs of meningeal irritation.

Investigations revealed normal hemoglobin (12.8 gm/dl), total leukocyte counts (6400/mm^3^) and differential leukocyte counts (neutrophils 54%, lymphocytes 44%, monocytes 1% and basophils 1%) and mild thrombocytopenia (100 × 10^3^/mm^3^). Blood biochemistry showed normal fasting sugar (108 mg/dl), urea (26 mg/dl), creatinine (0.8 mg/dl), calcium (8.6 mg/dl), sodium (142 mg/dl) and potassium (4.2 mg/dl). Liver function tests revealed mildly raised SGOT/PT (78/67 IU) and reversed albumin:globulin (3.8/4.9 gm/dl) ratio. Erythrocyte sedimentation rate was 45 mm in 1^st^ hour measured by Westergren’s method. Blood smear for malaria parasite was negative. Cultures from blood and urine were sterile. Widal test showed normal titers. Testing for viral markers (HIV I and II, HBs Ag and anti HCV) were negative. Chest radiograph did not show any abnormality. Contrast enhanced computer tomographic (CECT) scan of chest and abdomen revealed hepato-splenomegaly and an insignificant (<1 cm) peri-pancreatic lymph node in abdomen; no abnormality noticed in the chest.

Patient was on symptomatic treatment with antipyretics. During hospital stay patient noticed increased sleepiness and increased frequency and duration of tremors. Meanwhile, we contacted, telephonically, the laboratory at Nairobi West hospital, Kenya where patient’s blood tests were performed before returning to India. We were told that quantitative buffy coat (QBC) test (code no. 1021193, A/C No. 82609, Ex No. 144572) was positive for trypanosome. At our institute, repeat testing for trypanosome by direct smear, giemsa and QBC testing were negative. Cerebrospinal fluid (CSF) examination was acellular with normal glucose (93 mg/dl; corresponding blood glucose was 104 mg/dl). The proteins were raised (93 mg/dl) and globulins were positive. Examination of CSF for trypanosome by direct smear, giemsa and QBC were negative. Bone marrow aspirate did not reveal any organism. Bone biopsy showed single ill defined granuloma.

Magnetic resonance imaging (MRI) of brain showed mild meningeal enhancement, most probably post lumbar puncture enhancement. Few small lesions noticed in pons, which were hyperintense on T2-weighted and FLAIR images. Possibility of infective/inflammatory etiology was kept, though these could be non specific.

Considering the fact that disease is fatal without treatment and therapy is associated with significant morbidity, it was a great dilemma to treat HAT based on the telephonic reports from Kenya. However, we could not make alternate diagnosis. Therefore, we decided to start therapy for HAT. In view of our lack of experience, we consulted Dr. Ohennu Blum, WHO personnel, by e-mail for management of HAT. He kindly advised various regimens used for treating HAT. We arranged suramin and melarsoprol through WHO. We decided to treat sleeping sickness following the Tanzanian scheme for stage 2 trypanosomiasis. Patient received suramin, after 1.5 gm of suramin patient became afebrile on day 3, but noticed increased somnolence. A repeat CSF examination showed 5 WBC (lymphocyte)/µl; sugar and protein were normal and globulins were negative. Melarsoprol was added with intension to give three cycles (180 mg/day × 3 days) weekly for three weeks. However, on day 3 of first cycle we noticed worsening of tremor. Now patient became ataxic, although other neurological examination was normal. So, we repeated CSF examination which showed 10 lymphocytes/µl with raised proteins. MRI of brain showed multiple hyper-intense, minimally enhancing white matter lesions which were reported as nonspecific. We were continuously explaining the patient and his attendant about the daily progress and our limited experience of treating HAT. Patient’s attendants decided to take patient to University Hospital at Antwerp, Belgium for further management.

In Belgium, patient was further evaluated for cause of symptoms complex. Initially, tuberculosis was considered as first differential and brain biopsy was planned. However, investigations revealed serology positive for Brucella which was further confirmed by positive culture. Patient was started on rifampin, doxycycline plus co-trimoxazole based regimen for 6 months. He responded well to treatment. When last seen almost one year of completion of therapy was asymptomatic.

## Discussion

Human African trypanosomiasis and brucellosis both are zoonotic diseases. Brucellosis is endemic in countries bordering the Mediterranean Sea and many other countries [1]. Humans can acquire brucellosis by direct inoculation through cuts and skin abrasions, especially from handling animal tissues or secretions, via the conjunctiva, inhalation of infected aerosols and ingestion of contaminated food such as raw milk, cheeses made from unpasteurized milk, or raw meat. Clinically, brucellosis invariably presents with fever. Systemic involvement may affect musculoskeletal (osteomyelitis, arthritis or abscess), hepatosplenomegaly, genitourinary (epidydimo-orchitis), central nervous system (neurobrucellosis), cardiac (endocarditis) etc. The clinical spectrum of neurobrucellosis is very heterogeneous. The presentation may be with depression or lethargy or full blown meningitis, encephalitis, polyradiculoneuritis, cranial nerve involvement, epilepsy, brain abscess, subarachnoid hemorrhage and coma [2]. Examination of CSF reveals a lymphocytic reaction with a raised protein. The organism can be cultured from the CSF only in a minority of cases. In suspected cases diagnosis is confirmed by enzyme linked immunosorbant assay (ELISA) which is positive in blood and CSF [3].

Treatment of brucellosis includes combination of drugs which include tetracyclines, streptomycin, co-trimoxazole (TMP-SMX) and rifampicin. The aim the treatment is to treat current infection and prevention of relapse. Various regimens described are Streptomycin plus doxycycline, rifampicin *plus* doxycycline, or TMP-SMX with Streptomycin or rifampicin (in patients who can not take tetracyclines). Usual duration of treatment is 6-weeks. However, neurobrucellosis requires a prolonged therapy (3-6 months). With these drugs response rate is good with relapse/failure rate of about 10% [4,5]. Our patient was treated with rifampicin *plus* doxycycline for six months and responded well.

HAT is also a zoonotic disease (affecting both wild and domestic animals) caused by a protozoan, genus Trypanosome (gambiense and rhodesiense spp.). Humans are infected by the bite of Tsetse fly (Glossina spp.) which acts as a vector. The initial lesion is characterized by formation of chancre at site of tsetse fly bite in 5-10 days. There are two recognized stages in the clinical presentation of HAT - early hemolymphatic stage (early), and the late encephalitic stage (late). Early stage is characterized by episodes of fever (lasting 1-7 days) and generalized lymphadenopathy usually after 1-3 weeks of bite. Other symptoms include malaise, headache, arthralgia, lassitude, and weight loss [6]. Infection can involve organs like spleen, liver, skin, cardiovascular system, endocrine system, and eyes [7]. Late stage is characterized by neuropsychiatric manifestations. The psychiatric symptoms include irritability, lassitude, headache, personality changes, hallucinations, suicidal tendencies, and mania [7,8]. Neulogical manifestations can be motor (limb tremors, tongue and limb muscle fasciculation, limb hypertonia and pyramidal weakness, choreiform and athetoid movements, dysarthria, cerebellar ataxia, and polyneuritis) or sensory (painful hyperaesthesia, pruritus, and deep hyperaesthesia i.e. Kerandel’s sign) [7-9]. Sleep disturbances are characterized by lassitude, distractibility, spontaneous, uncontrollable urges to sleep and a reversal of the normal sleep-wake cycle. In absence of treatment disease progresses to the final stage characterized by seizures, severe somnolence, double incontinence, cerebral edema, coma, systemic organ failure, and death [7-9].

The first clue to diagnosis of HAT is a clinical presentation in the context of a geographical location or history of travel to an endemic area. The definite diagnosis involves demonstration of the trypanosomes in the peripheral blood using stained thick and thin films or in other infected tissues such as lymph node or bone marrow aspirates. Antibody-detecting card agglutination test is a simple, easy and rapid test for serological diagnosis [10]. However, the most crucial part in HAT diagnosis and therapeutic decision making is to distinguish reliably the late encephalitic stage of HAT from the early stage as failure to treat a patient with CNS involvement will inevitably lead to death from the disease and inappropriate CNS treatment in an early-stage patient carries a high risk of unnecessary drug toxicity. In patients with suspected late-stage disease it is imperative to perform a lumbar puncture, which typically shows a lymphocytic pleiocytosis and raised protein level of 40-200 mg/100 ml [8]. There is lack of a universal consensus on the operational definition of late-stage HAT. WHO criteria for encephalytic stage are demonstration of the parasites in the CSF or a white blood cell (WBC) count of >5/µl. Our patient’s CSF parameters (10 lymphocytes/µl and raised proteins) were confused with encephalitic stage of HAT. Recently, it has been suggested that the WHO criteria should be replaced by the presence of intrathecal IgM synthesis or the presence of >20 WBCs/µl, independent of the presence of trypanosomes in the CSF [11]. EEG can show non specific changes. MRI of the brain may show diffuse asymmetric white matter abnormalities, diffuse hyper-intensities in the basal ganglia, and ventricular enlargement [12]. Our case also demonstrated multiple hyper-intense white matter lesions. CSF PCR is another test which has sensitivity of about 95% but currently not used for therapeutic decision making [13].

Current therapy for HAT is based on four drugs namely suramin, pentamidine, melarsoprol, and eflornithine. Early-stage disease is treated with i.v. suramin in rhodesiense disease and with intramuscular (i.m.) pentamidine in gambiense disease. Melarsoprol is the only effective drug for late-stage disease in both forms of HAT [14]. A course of 3-4 i.v. doses are given daily over a week for a total period of 3-4 weeks. About 80-90% patients are cured with standard regimens [8]. Major problem with melarsoprol treatment is a severe post-treatment reactive encephalopathy (PTRE) in up to 10% of cases, with a fatality rate of about 50% [12]. Simultaneous use of prednisolone can reduce this complication.

Our case, probably do not add much to the exiting clinical knowledge, however, it highlights the impact of globalization on patients and medical professionals. In this era, both diseases as well as medical professional expertise do not respect the global boundaries. It has enabled us to share our knowledge and experiences with our professional colleagues over the globe within short time. It takes only few hours to days to take professional opinion or medical aids across the world. Similarly, it has led us to study more closely about the diseases/conditions which are rarely seen in that particular part of the globe. However, it has its dark side also. As lack of experience of management of the disease in question may be a problem. First, we may miss diagnosis during differential diagnoses. Secondly, even if we have considered the possibility of some ‘alien’ disease then there are high chances of lacking facility to rule out or confirm that possibility. Finally, it may be difficult to treat any disease in absence of experience. Like our case was treated wrongly as HAT. Serological test demonstrating antibody response against trypanosome could have been an option to rule out past infection however, this facility is not available at our institute. Finally, with the help of our professional colleagues in Belgium possibility trypnosomal infection (past or active) was ruled out and diagnosis of brucellosis made and treated successfully.

## Conclusion

Globalization has affected whole world, including medical professionals, in significant and positive way. It has enabled us to exchange our professional knowledge and expertise across the globe for better care to the patients in need. Brucellosis is a frequently missed cause of pyrexia of unknown. However, if suspected, diagnosis is easy and treatment is usually satisfactory.

## References

[bib-001] Young EJ, Mandell GL, Douglas RG, Bennett JE (2005). Brucella Species. Principles and Practice of Infectious Diseases.

[bib-002] Samdani PG, Patil S (2003). Neurobrucellosis. Indian Pediatr.

[bib-003] Shakir RA (1986). Neurobrucellosis. Postgrad Med J.

[bib-004] Hasanzani Roushan MR, Mohraz M, Hajiahmadi M, Ramzani A, Valayati AA (2006). Efficacy of gentamycin and doxycycline versus streptomycin plus doxycycline in the treatment of brucellosis in humans. Clin Infect Dis.

[bib-005] Pappas G, Solera J, Akritidis N, Tsianos E (2005). New approaches to the antibiotic therapy of brucellosis. Int J Antimicrob Agents.

[bib-006] Apted FIC, Mulligan HW (1970). Clinical manifestations and diagnosis of sleeping sickness. The African trypanosomiasis.

[bib-007] Duggan AJ, Hutchington MP (1966). Sleeping sickness in Europeans: a review of 109 cases. J Trop Med Hyg.

[bib-008] Atouguia JLM, Kennedy PGE, Davis LE, Kennedy PGE (2000). Neurological aspects of human African trypanosomiasis. Infectious diseases of the nervous system.

[bib-009] Kristensson K, Grassi-Zucconi G, Bentivoglio M, Rose FC (1995). Nervous system dysfunctions in African trypanosomiasis. Recent advances in tropical neurology.

[bib-010] Truc P, Lejon V, Magnus E, Jamonneau V, Nangouma A, Verloo D, Penchenier L, Büscher P (2002). Evaluation of the micro-CATT, CATT/Trypanosoma brucei gambiense, and LATEX/T.b. gambiense methods for serodiagnosis and surveillance of human African trypanosomiasis in West and Central Africa. Bull World Health Organ.

[bib-011] Lejon V, Reiber H, Legros D, Djé N, Magnus E, Wouters I, Sindic CJ, Büscher P (2003). Intrathecal immune response pattern for improved diagnosis of central nervous system involvement in trypanosomiasis. J Infect Dis.

[bib-012] Gill DS, Chatha DS, del Carpio-O’Donovan R (2003). MR imaging findings in African trypansomiasis. Am J Neuroradiol.

[bib-013] Jamonneau V, Solano P, Garcia A, Lejon V, Djé N, Miezan TW, N’Guessan P, Cuny G, Büscher P (2003). Stage determination and therapeutic decision in human African trypanosomiasis: value of polymerase chain reaction and immunoglobulin M quantification on the cerebrospinal fluid of sleeping sickness patients in Côted’Ivoire. Trop Med Int Health.

[bib-014] Pepin J, Milord F (1994). The treatment of human African trypanosomiasis. Adv Parasitol.

